# Socioeconomic status and mortality after acute myocardial infarction: a study from Iran

**DOI:** 10.1186/1475-9276-10-9

**Published:** 2011-02-07

**Authors:** Tahereh Donyavi, Kourosh Holakouie Naieni, Saharnaz Nedjat, Mariam Vahdaninia, Mahdi Najafi, Ali Montazeri

**Affiliations:** 1 School of Public Health, Tehran University of Medical Sciences, Tehran, Iran; 2 Department of Social Medicine, Iranian Institute for Health Sciences Research, ACECR, Tehran, Iran; 3 Department of Anesthesiology, Tehran Heart Centre, Tehran University of Medical Sciences, Tehran, Iran; 4 Department of Mental Health, Iranian Institute for Health Sciences Research, ACECR, Tehran, Iran

## Abstract

**Background:**

Studies have shown an inverse relationship between socioeconomic status (SES) and mortality due to coronary heart disease (CHD). Little is known about this association in Iran. This study aimed to investigate whether mortality after myocardial infarction (MI) varies by SES.

**Methods:**

In a retrospective study, 1283 MI patients who hospitalized in Tehran Heart Center from March 2005 to March 2006 were followed up in March 2008. Demographic, clinical and SES data were collected from case records and by telephone interviews. Multiple logistic regression analysis was performed to estimate the predictive effect of socioeconomic factors on outcome.

**Results:**

In all 664 patients were studied. Of these, 500 patients were alive and 164 were dead due to MI (64 died at hospital and 100 died at home). The results of regression analysis showed that in addition to treatment (OR = 9.52, 95%CI 4.84-18.7), having diabetes (OR = 1.78, 95% CI 1.12-2.81) or hyperlipidemia (OR = 1.82, 95% CI 1.14-2.90), socioeconomic variables including living area in square per person (lowest level vs. upper level OR = 4.92, 95% CI 2.11-11.4), unemployment (OR = 3.50, 95% CI 1.50-8.13) and education (OR for illiterate patients = 2.51, 95% CI 1.00-6.31) were the most significant contributing factors to increased mortality after MI.

**Conclusion:**

Although the findings should be interpreted with caution, the study results indicated that socioeconomic variables were significant contributing factors to increased mortality after myocardial infarction. The underlying role of socioeconomic status on increased mortality after MI deserves further investigation.

## Background

Coronary heart disease (CHD) is the first killer of Iranian population. Annually there are about 138,000 deaths due to CHD (about 40% of total deaths). A bout 50% of deaths occur due to myocardial infarction [1]. It is a leading cause of morbidity and disability in Iranian population [2]. Since no efficient referral system exists in Iran, people with heart diseases directly attend to private sector, clinics or state hospitals and seen by internal medicine specialist or cardiologist. Patients with myocardial infarction (MI) usually attend to private, state or teaching hospitals as emergency admissions. According to patients' medical coverage they should pay for their care. There is evidence that the number of patients with MI are increasing and during a five-year period for instance cardiac surgery increased by 80% in a teaching hospital [3]. Recently, Iranian ministry of health implemented different preventive measures including creating different centers for cardiovascular diseases control.

In addition to known risk factors for CHD [4] it appears that people's socioeconomic status also contributes to the outcome. The association between socioeconomic position and outcome of myocardial infarction (MI) is generally well documented in western countries indicating that those with lower socioeconomic status experience the most burden of the condition [5-10]. A historical study among British civil servants in 1981 found that social class (as measured by occupation) was a significant contributing factor to increased risk of CHD while age, smoking, height, body mass index, systolic blood pressure, cholesterol and blood glucose showed only a moderate impact [11]. Similarly a study from the USA found that disadvantaged acute MI patients receive fewer specialized procedures and therefore show higher mortality due to CHD [12].

Much attention has been paid to how socioeconomic status (SES) might play a role on the outcome of CHD. There has been a debate if geographical service patterns and accessibility to medical care are responsible for such an association. For instance a study from Canada found that geography and service supply do not explain socioeconomic gradients in angiography use after acute myocardial infarction [13]. Further analysis of the same study indicated that upper middle-class Canadians gain preferential access to services within the publicly funded health care system as compared to those with lower incomes or less educated individuals [14]. However, consequent studies from the same country showed that there were geographical barriers to cardiac catheterisation and MI patients who live outside of metropolitan area and they had lower rates of cardiac catheterisation, longer waiting times and increased rate of readmission, and thus poorer outcomes [15].

Little is known about the association of SES and outcome of CHD in developing countries such as Iran. However recently the Isfahan Cardiovascular Research Centre (a WHO collaborating center for research and training in cardiovascular disease control in central Iran) carried out an analysis of available data of 12514 individuals and found that socioeconomic factors as measured by education, occupation and income were associated with cardiovascular risk factors [16]. The study reported mixed results and did not indicate the association between the adverse outcome of the disease and socioeconomic variables.

The main objective of this study was to investigate whether socioeconomic variables independently contribute to excess mortality after myocardial infarction in Iran. We thought the findings from this first study could contribute to existing literature on the topic and also could provide evidence for better understanding of social determinants of health in general and the issue of health equity in particular in Iran and perhaps in other developing countries of similar conditions.

## Methods

### Design

This was a retrospective study of a cohort of 1283 MI patients who admitted to Tehran Heart Center, a large teaching and referral hospital affiliated to Tehran University of Medical Sciences between March 2005 and March 2006 (one complete Iranian calendar year). All case records were reviewed and patients' clinical records were extracted. Patients were followed-up in March 2008 in order to find out whether they were still alive. One of us contacted all patients' by telephone. In each successful contact either patient or a close relative (wife, husband, son or daughter, parents and sister or brother) were interviewed. For each unsuccessful contact two extra attempts were made and if still could not find the patient, it was regarded as missing.

### Demographic information

Demographic data included recording of age, gender, marital status, and employment.

### Clinical information

This included information on body mass index (BMI), and known risk factors for myocardial infarction. The risk factors were: history of high blood pressure (blood pressure equal of greater than 130/85 mmGH), diabetes (fasting blood glucose equal or greater than 126 mg/dl), hyperlipidemia (cholesterol equal or greater than 200 mg/dl and triglyceride equal or grater than 150 mg/dl), and smoking. BMI was categorized as recommended by the WHO: normal range (BMI = 18.5-24.9 kg/m^2^), overweight (BMI = 25-29.9 kg/m^2^), and obese (BMI ≥ 30 kg/m^2^) [17]. Information regarding treatment also was collected. Patients received either invasive (coronary artery by pass graft) or non-invasive (pharmacologic) treatments. Non-surgical treatment included prescriptions of aspirin, beta-adrenergic blocking agents (β-blockers) and angiotensin-converting enzyme (ACE) inhibitors. The decision regarding mode of therapy depended upon the individual patient's prognosis as determined by both cardiac and non-cardiac conditions. In general patients who were at higher risk as defined by the severity of angina and/or ischemia, the number of diseased vessels, and the presence of left ventricular dysfunction received surgical treatment. Data were extracted from case records.

### Socioeconomic measures

We used years of formal education as a measure for social position. There is evidence that it is a valid and reliable indicator for studies of association between health and social status in Iran [18]. Education was categorized into five levels: no education, first level (1 to 5 years), second level (6-9 years), third level (10-12 years) and fourth level (more than 12 years). Economic status was assessed by average living area in square per person (m^2^/p). This proxy measure was categorized into four levels: less than 10, 10-19, 20-39, and equal or more than 40 square per person living area. Since income information in Iran is not reliable and people usually have more than one job at the same time, we did not collect or use data on income as a measure of socioeconomic position. Years of education were extracted from case records but average living area per person was calculated based on information collected during interviews.

### Statistical analysis

In addition to descriptive statistics, multiple logistic regression analysis was performed to calculate odds ratios (ORs) and to examine the predictive effect of socioeconomic and clinical variables on risk for mortality after myocardial infarction. For the analyses patients' status (alive vs. dead) was considered as independent variable. Socio-demographic and clinical information were regarded as dependent factors and all were entered into the model. We carried out analyses for in-hospital and out-of-hospital deaths separately, in addition to presenting the combined analyses. All the deaths examined in the present study were cardiac deaths.

## Results

### Patients' characteristics

In all 1283 patients were admitted to the Tehran Heart Center between March 2005 and March 2006. After a two-year fallow-up only 664 patients were available for the study. Of these, 500 patients were alive and 164 were dead due to myocardial infarction (65 died at hospital and 99died at home). The socio-demographic and clinical characteristics of patients are presented in Table 1 and Table 2.

**Table 1:** Demographic and clinical characteristics of the study population

	Study population (n = 1283)	Study sample (n = 664)	Drop-out sample (n = 619)	
	**No. (%)**	**No. (%)**	**No. (%)**	**P**

**Age (years)**				0.15

≤ 49	214 (16.7)	105 (15.8)	109 (17.6)	

50-59	304 (23.7)	144 (21.7)	160 (25.8)	

60-69	363 (28.3)	193 (29.1)	170 (27.5)	

≥ 70	402 (31.3)	222 (33.4)	180 (29.1)	

Mean (SD)	62.4 (12.3)	63.0 (12.0)	61.7 (12.7)	0.05*

Range	21-103	21-91	26-103	

**Gender**				0.003

Male	950 (74.0)	468 (70.5)	482 (77.9)	

Female	333 (26.0)	196 (29.5)	137 (22.1)	

**Marital status**				0.11

Single	42 (3.3)	24 (3.6)	18 (2.9)	

Married	1024 (79.8)	515 (77.6)	509 (82.2)	

Widowed/divorced	217 (16.9)	125 (18.8)	92 (14.9)	

**Education (years)**				0.95

0	337 (26.3)	177 (26.7)	160 (25.8)	

1-5	285 (22.3)	145 (21.8)	140 (22.6)	

6-9	313 (24.4)	160 (24.1)	153 (24.7)	

10-12	218 (16.9)	111 (16.7)	107 (17.2)	

> 12	130 (10.1)	71 (10.7)	59 (9.5)	

**Employment**				0.06

Housewife	282 (22.0)	157 (23.6)	125 (20.2)	

Employed	615 (47.9)	297 (44.7)	318 (51.4)	

Unemployed	115 (9.0)	68 (10.2)	47 (7.6)	

Retired	271 (21.1)	142 (21.4)	129 (20.8)	

**BMI (kg/m**^**2**^)*				0.25

≤ 24.9	362 (28.3)	180 (27.1)	182 (29.4)	

25-29.9	728 (56.7)	374 (56.3)	354 (57.2)	

≥ 30	193 (15.0)	110 (16.6)	83 (13.4)	

**Hypertension**				0.44

No	703 (54.8)	357 (53.8)	346 (55.9)	

Yes	580 (45.2)	307 (46.2)	273 (44.1)	

**Diabetes**				0.99

No	833 (64.9)	431 (64.9)	402 (64.9)	

Yes	450 (35.1)	233 (35.1)	217 (35.1)	

**Hyperlipidemia**				0.43

No	601 (46.8)	304 (45.8)	297 (48.0)	

Yes	682 (53.2)	360 (54.2)	322 (52.0)	

**History of smoking**				0.03

No	416 (32.4)	198 (29.8)	218 (35.2)	

Yes	867 (67.6)	466 (70.2)	4.01 (64.8)	

**Treatment**				< 0.0001**

Coronary artery bypass graft	333 (26.0)	232 (34.9)	101 (16.3)	

Non-surgical	950 (74.0)	432 (65.1)	518 (83.7)	

* t-test

** Fisher's exact test

**Table 2:** Demographic and clinical characteristics of the study sample (n = 664)

	Discharged (n = 500)	Dead (n = 164)	
	**No. (%)**	**No. (%)**	**P**

**Age (years)**			<0.0001

≤ 49	90 (18.0)	15 (9.1)	

50-59	121 (24.2)	23 (14.0)	

60-69	152 (30.4)	41 (25.0)	

≥ 70	137 (27.4)	85 (51.8)	

Mean (SD)	61.5 (11.6)	67.6 (12.1)	< 0.0001*

Range	21-88	29-91	

**Gender**			< 0.0001

Male	374 (74.8)	94 (57.3)	

Female	126 (25.2)	70 (42.7)	

**Marital status**			< 0.0001

Single	21 (4.2)	3 (1.8)	

Married	406 (81.2)	109 (66.5)	

Widowed/divorced	73 (14.6)	52 (31.7)	

**Education (years)**			< 0.0001

0	104 (20.8)	73 (44.5)	

1-5	107 (21.4)	38 (23.2)	

6-9	132 (26.4)	28 (17.1)	

10-12	94 (18.8)	17 (10.4)	

> 12	63 (12.6)	8 (4.9)	

**Employment**			< 0.0001

Housewife	108 (21.6)	49 (29.9)	

Employed	249 (49.8)	48 (29.3)	

Unemployed	30 (6.0)	38 (23.2)	

Retired	113 (22.6)	29 (17.7)	

**Living area in square per person (m**^**2**^**/p)**			0.001

< 10	52 (10.4)	29 (17.7)	

10-19	186 (37.2)	78 (47.6)	

20-39	171 (34.2)	41 (25.0)	

≥ 40	91 (18.2)	16 (9.8)	

**BMI (kg/m**^**2**^)*			0.06

≤ 24.9	145 (29.0)	35 (21.4)	

25-29.9	269 (53.8)	105 (64.0)	

≥ 30	86 (17.2)	24 (14.6)	

**Hypertension**			0.04

No	280 (56.0)	77 (47.0)	

Yes	220 (44.0)	87 (53.0)	

**Diabetes**			0.001

No	343 (68.6)	88 (53.7)	

Yes	157 (31.4)	76 (46.3)	

**Hyperlipidemia**			0.10

No	238 (47.6)	66 (40.2)	

Yes	262 (52.4)	98 (59.8)	

**History of smoking**			0.01

No	162 (32.4)	36 (22.0)	

Yes	338 (67.6)	128 (78.0)	

**Treatment**			< 0.0001

Coronary artery bypass graft	221 (44.2)	11 (6.7)	

Non-surgical	279 (55.8)	153 (93.3)	

* t-test

### Outcome analysis

Using patients' status (alive or dead) as outcome variable, we performed multiple logistic regression analysis while entering patients' socio-demographic and clinical characteristics as independent variables in the model. The results for in- and out-of-hospital deaths are shown in Table 3 and Table 4 separately. The combined results are shown in Table 5. The findings indicated that in addition to treatment (OR = 9.52, 95%CI 4.84-18.7), having diabetes (OR = 1.78, 95% CI 1.12-2.81) or hyperlipidemia (OR = 1.82, 95% CI 1.14-2.90), socioeconomic variables including living area in square per person (lowest level vs. upper level OR = 4.92, 95% CI 2.11-11.4), unemployment (OR = 3.50, 95% CI 1.50-8.13) and education (OR for illiterate patients = 2.51, 95% CI 1.00-6.31) were the most significant contributing factors to increased mortality after myocardial infarction.

**Table 3:** The results obtained from multiple logistic regression analysis indicating risk factors for mortality after myocardial infarction for in-hospital deaths (n = 500 alive vs. 65 in-hospital deaths)

		OR (95% CI)	P
**Age**			

	≤ 49	1.00 (ref.)	

	50-59	0.41 (0.09-1.88)	0.25

	60-69	0.59 (0.16-2.22)	0.44

	≥ 70	1.66 (0.45-6.11)	0.44

**Gender**			

	Male	1.00 (ref.)	

	Female	0.89 (0.22-3.63)	0.87

**Marital status**			

	Married	1.00 (ref.)	

	Never married	4.85 (0.48-49.0)	0.18

	Widowed/divorced	1.98 (0.81-4.79)	0.12

**Education (years)**			

	> 12	1.00 (ref.)	

	10-12	0.74 (0.53-2.48)	0.66

	6-9	0.69 (0.34-2.30)	0.81

	1-5	1.18 (0.45-3.07)	0.72

	Illiterate	1.51 (0.87-6.31)	0.14

**Employment**			

	Employed	1.00 (ref.)	

	Unemployed	12.05 (3.6-43.1)	<0.001

	Retired	3.28 (1.04-10.30)	0.04

	Housewife	2.54 (0.47-13.68)	0.27

**Living area in square per person (m**^**2**^**/p)**			

	≥ 40	1.00 (ref.)	

	20-39	5.22 (1.24-21.9)	0.02

	10-19	16.58 (3.50-78.54)	<0.001

	< 10	16.78 (4.19-67.1)	<0.001

**BMI (kg/m**^**2**^**)**			

	≤ 24.9	1.00 (ref.)	

	25-29.9	1.64 (0.44-6.15)	0.45

	≥ 30	3.78 (1.41-10.09)	0.008

**Hypertension**			

	No	1.00 (ref.)	

	Yes	1.17 (0.83-1.75)	0.62

**Diabetes**			

	No	1.00 (ref.)	

	Yes	1.59 (0.78-3.27)	0.20

**Hyperlipidemia**			

	No	1.00 (ref.)	

	Yes	1.33 (0.62-2.85)	0.45

**History of smoking**			

	No	1.00 (ref.)	

	Yes	2.01 (0.79-5.09)	0.13

**Treatment**			

	Coronary artery bypass graft	1.00 (ref.)	

	Non-surgical	10.24 (3.16-33.15)	<0.001

**Table 4:** The results obtained from multiple logistic regression analysis indicating risk factors for mortality after myocardial infarction for out-of-hospital deaths (n = 500 alive vs. 99 out-of-hospital deaths)

		OR (95% CI)	P
**Age**			

	≤ 49	1.00 (ref.)	

	50-59	1.47 (0.58-3.73)	0.41

	60-69	1.36 (0.53-3.45)	0.51

	≥ 70	1.86 (0.72-4.77)	0.19

**Gender**			

	Male	1.00 (ref.)	

	Female	3.83 (1.26-11.59)	0.01

**Marital status**			

	Married	1.00 (ref.)	

	Never married	0.99 (0.19-5.08)	0.99

	Widowed/divorced	0.60 (0.27-1.31)	0.20

**Education (years)**			

	> 12	1.00 (ref.)	

	10-12	0.60 (0.20-1.83)	0.37

	6-9	0.61 (0.22-1.72)	0.35

	1-5	1.01 (0.37-2.76)	0.98

	Illiterate	1.82 (0.69-4.78)	0.22

**Employment**			

	Employed	1.00 (ref.)	

	Unemployed	1.27 (0.62-2.57)	0.50

	Retired	0.95 (0.28-3.20)	0.93

	Housewife	0.41 (0.12-1.35)	0.14

**Living area in square per person (m**^**2**^**/p)**			

	≥ 40	1.00 (ref.)	

	20-39	1.78 (0.81-3.92)	0.15

	10-19	2.56 (1.18-5.52)	0.01

	< 10	3.25 (1.27-8.29)	0.01

**BMI (kg/m**^**2**^**)**			

	≤ 24.9	1.00 (ref.)	

	25-29.9	1.05 (0.56-1.78)	0.98

	≥ 30	1.06 (0.47-2.11)	0.99

**Hypertension**			

	No	1.00 (ref.)	

	Yes	1.16 (0.68-1.98)	0.57

**Diabetes**			

	No	1.00 (ref.)	

	Yes	1.92 (1.13-3.27)	0.01

**Hyperlipidemia**			

	No	1.00 (ref.)	

	Yes	2.16 (1.24-3.74)	0.006

**History of smoking**			

	No	1.00 (ref.)	

	Yes	1.10 (0.58-2.10)	0.75

**Treatment**			

	Coronary artery bypass graft	1.00 (ref.)	

	Non-surgical	9.64 (4.26-21.83)	<0.001

**Table 5:** The results obtained from multiple logistic regression analysis indicating risk factors for mortality after myocardial infarction for all deaths (n = 500 alive vs. 164 in- and out-of-hospital deaths combined)

		OR (95% CI)	P
**Age**			

	≤ 49	1.00 (ref.)	

	50-59	1.12 (0.49-2.52)	0.78

	60-69	1.18 (0.53-2.59)	0.68

	≥ 70	1.97 (0.89-4.36)	0.09

**Gender**			

	Male	1.00 (ref.)	

	Female	2.22 (0.86-5.69)	0.09

**Marital status**			

	Married	1.00 (ref.)	

	Never married	1.47 (0.36-5.95)	0.58

	Widowed/divorced	0.92 (0.50-1.72)	0.06

**Education (years)**			

	> 12	1.00 (ref.)	

	10-12	0.91 (0.33-2.48)	0.86

	6-9	0.89 (0.34-2.30)	0.81

	1-5	1.18 (0.45-3.07)	0.72

	Illiterate	2.51 (1.00-6.31)	0.04

**Employment**			

	Employed	1.00 (ref.)	

	Unemployed	3.50 (1.50-8.13)	0.004

	Retired	1.18 (0.79-2.77)	0.21

	Housewife	0.67 (0.23-1.94)	0.46

**Living area in square per person (m**^**2**^**/p)**			

	≥ 40	1.00 (ref.)	

	20-39	2.34 (1.14-4.79)	0.01

	10-19	4.38 (2.19-8.75)	< 0.0001

	< 10	4.92 (2.11-11.4)	< 0.0001

**BMI (kg/m**^**2**^**)**			

	≤ 24.9	1.00 (ref.)	

	25-29.9	1.43 (0.85-2.41)	0.17

	≥ 30	1.09 (0.55-2.17)	0.78

**Hypertension**			

	No	1.00 (ref.)	

	Yes	1.05 (0.66-1.66)	0.81

**Diabetes**			

	No	1.00 (ref.)	

	Yes	1.78 (1.12-2.81)	0.01

**Hyperlipidemia**			

	No	1.00 (ref.)	

	Yes	1.82 (1.14-2.90)	0.01

**History of smoking**			

	No	1.00 (ref.)	

	Yes	1.33 (0.77-2.31)	0.30

**Treatment**			

	Coronary artery bypass graft	1.00 (ref.)	

	Non-surgical	9.52 (4.84-18.7)	< 0.0001

## Discussion

This study investigated whether survival after MI varied with socio-economic status and analyzed a two-years mortality rate among 664 MI patients who hospitalized in Tehran Heart Center during a one complete calendar year. The results obtained from regression analysis adjusted for demographic, clinical and socio-economic variables showed that education was a predictor of mortality among patients with MI. The lower educational level group (illiterate and primary) showed higher mortality risk compared to the higher level group. The association between education and mortality due to MI is well documented where studies showed that patients with lower educational levels experience lower survival rates after an acute MI attack [19,20]. In addition there is evidence that years of education can strongly contribute in the distribution of several risk factors for CHD such as smoking and HBP that may well inversely have an effect on the incidence and impact of MI attack [21].

The results also indicated that unemployed and retired individuals showed a higher risk of mortality after MI (P = 0.004). Earlier surveys have found income as an independent factor for outcome following MI. In fact, lower income was associated with increased short and long-term mortality [22,23]. Even though a study from Canada has stated that the effect of income on mortality rate due to MI can be modified by age, past cardiovascular events and current vascular risk factors [24].

Economic status as measured by 'living area in square per person' demonstrated a strong correlation with mortality caused by MI. It was found that people who were living in larger places have experienced decreased mortality after MI. Similarly in a follow-up study in Sweden it was revealed that socioeconomic environment plays an essential role in the survival rate of patients with MI and worse long term prognosis is expected for patients from less affluent residential areas [25]. A study investigated the hypothesis that how the neighbourhood status may link to cardiovascular risk factors and excess mortality rate. This study was carried out in nine industrial towns from Czech Republic and Germany and pointed out that area level socioeconomic is associated with health related lifestyles which might be a possible pathway linking social status and cardiovascular diseases [26].

Receiving non-surgical treatment was a significant prognostic factor for higher mortality. We do not know the exact reasons for not receiving surgical treatment; nevertheless we do speculate that patients' economic status was one of the most possible reasons. Evidence suggests that financial difficulties among patients with MI are the main factors that can cause poorer outcome and elevated possibility of re-hospitalization [27]. A strong association was reported between early recurrent ischaemic occurrences and SE deprivation that was not accounted for by clinical presentation or treatment [28]. However, there might be two other additional explanations for such an observation: the patients did not have medical indications for surgery, and that patients themselves preferred other treatments. Future studies should clearly ask patients about this in order to indicate whether they could not afford surgery or irrespective of financial issues they received other treatments.

## Limitations

This study has several limitations. The most important concern is that there was a large-drop-out. Studies have shown a higher mortality risk in non-responders to a baseline survey adjusted for different measures of patients' socio-economic status. For instance Harald et al. [29] found a 2-fold higher mortality risk in non-responders to baseline in every socioeconomic category. Similarly Ferrie et al. [30] showed that non-responder to baseline had a mortality hazard double that for responders. Secondly, this study only included a patient population admitted to a single hospital. Earlier studies from other countries have reported rather large socioeconomic differences in mortality outside hospital (before reaching the hospital) and in the current study little can be said about them. It is recommended that the future studies should also investigate about patients who die before hospital admission. Thirdly, since the data was collected from one hospital, the results could not be generalized to the whole country. However, we believe the findings from this study are accurate since information presented here were extracted or collected with caution. In general the completeness and accuracy of case records in Iran (especially in teaching hospitals) are relatively good [31,32]. In addition studies have shown that information regarding demographic and socio-economic variables such as age, marital status, education or living conditions that are collected by interviews are valid [18,33].

In general the findings of current survey provided obvious evidence of the converse relationship between socio-economic variables and mortality after MI in Iranian patients. Coronary heart conditions are a main cause of mortality in Iranian population and can cause major work absenteeism [1,34]. The burden of CHD on the Iranian oil industry in 1999 to 2000 was examined and its direct cost was estimated about 22,770 million Iranian Rials (10,000 Rls. = 1 US$) [34]. Taking into consideration the underlying factors on high fatality due to CHD in Iran might have an essential role in understanding alleviating burden of the situation. Therefore, these findings may serve as possible recommendation for Iranian health policy.

## Conclusion

Although the findings should be interpreted with caution, the study results indicated that socioeconomic variables were significant contributing factors to increased mortality after myocardial infarction. The underlying role of socioeconomic status on increased mortality after MI should be further studied.

## Competing interests

The authors declare that they have no competing interests.

## Authors' contributions

TD was the main investigator, and collected the data. KH contributed to the study design and analysis. SN contributed to statistical analysis. MV contributed to writing process. MN advised on clinical aspects of the study and contributed to the discussion. AM initiated the study, analyzed the data and wrote the final draft. All authors read and approved the manuscript.
